# Simple, sensitive and specific quantification of diamine oxidase activity in complex matrices using newly discovered fluorophores derived from natural substrates

**DOI:** 10.1007/s00011-020-01359-5

**Published:** 2020-06-01

**Authors:** Thomas Boehm, Matthias Karer, Elisabeth Gludovacz, Karin Petroczi, Marlene Resch, Kornelia Schuetzenberger, Kristaps Klavins, Nicole Borth, Bernd Jilma

**Affiliations:** 1 Department of Clinical Pharmacology, Medical University of Vienna, Waehringer Guertel 18-20, 1090 Vienna, Austria; 2 Department of Biotechnology, University of Natural Resources and Life Sciences, Muthgasse 18, 1190 Vienna, Austria; 3 Center for Medical Physics and Biomedical Engineering, Medical University of Vienna, Waehringer Guertel 18-20, 1090 Vienna, Austria; 4 CeMM Research Centre for Molecular Medicine of the Austrian Academy of Sciences, Lazarettgasse 14, 1090 Vienna, Austria

**Keywords:** Diamine oxidase, Ortho-aminobenzaldehyde, Fluorophore, Quinazoline

## Abstract

**Objective:**

To measure diamine oxidase (DAO) activity with high sensitivity in complex matrices like plasma or tissue extracts radioactive putrescine or horseradish peroxidase (HRP)/hydrogen peroxide (H_2_O_2_) coupling must be used. The use of radioactive material should be avoided and HRP/H_2_O_2_ coupling is compromised by antioxidants.

**Methods and results:**

Condensation of ortho-aminobenzaldehyde (oABA) with delta-1-pyrroline and delta-1-piperideine, the autocyclization products of the DAO-oxidized natural substrates putrescine and cadaverine, generates new quinazoline fluorophores with absorption and excitation maxima of 430 and 460 nm, respectively, and peak emission at 620 nm. Fluorescent-based detection limits are 20–40 times lower compared to absorption measurements. This assay can be used to measure DAO activity in human plasma after spiking recombinant human (rh)DAO, in rat plasma after intravenous rhDAO administration, in pregnancy plasma and in tissue extracts of DAO wild-type and knock-out mice. Using rat plasma the correlation between rhDAO activity and ELISA data is 99%. Human and rat plasma without DAO spiking and tissue extracts from DAO knock-out mice showed stable and low fluorescence in the presence of high substrate concentrations.

**Conclusions:**

Incubation of DAO with the natural substrates putrescine and cadaverine and oABA generates novel fluorophores increasing the detection limit compared to absorption measurements at least tenfold. This simple, sensitive and specific assay allows the non-radioactive quantification of DAO activity in complex matrices like plasma and tissue extracts without interference by antioxidants.

**Electronic supplementary material:**

The online version of this article (10.1007/s00011-020-01359-5) contains supplementary material, which is available to authorized users.

## Introduction

Human diamine oxidase (E.C. 1.4.3.6) is a copper-containing amine oxidase that oxidatively deaminates histamine and various polyamines releasing ammonia and hydrogen peroxide [1]. It is an important enzyme in the extracellular catabolism of histamine and is highly expressed only in human intestine, kidney and fetal extravillous trophoblast cells [2–4].

For many decades the state of the art assay to measure DAO activity was based on autocyclization of radioactively-labelled putrescine or cadaverine followed by organic extraction and radioactivity measurements [5]. This assay is sensitive and specific, but working with radioactivity is expensive and not welcomed in the modern laboratory environments. It was also possible to measure serum DAO activity in non-pregnant healthy individuals [6]. A commercial assay using tritiated putrescine is available, but not suitable for DAO activity quantifications. We measured poor linearity using recombinant human (rh) DAO, possibly because the substrate concentration is several thousand-fold below the K_m_ to limit radioactivity exposure [7]. Lower DAO activity might be measured in tissue extracts with high capacity to convert the putrescine oxidation product gamma-aminobutyraldehyde into water soluble gamma-aminobutyric acid [8]. Bardsley [9] used para-dimethylaminomethylbenzylamine as DAO substrate but the affinity of this substrate is low [1] and the assay is not suitable for complex matrices, because the resulting aldehyde is directly measured at 250 nm and at this wavelength many interfering substances are present in tissue extracts or plasma.

In 1936 it was described that the condensation of ortho-aminobenzaldehyde (oABA) and delta-1-pyrroline, the autocyclized DAO oxidation product of putrescine, generates a yellow-orange chromophore [10]. This reaction can be used to measure DAO activity [11]. Nevertheless, the sensitivity is relatively low, because the extinction coefficient of the quinazoline chromophore is only 1860 M^−1^ cm^−1^ and many substances interfere in complex matrices like tissue extracts or plasma at the absorption peak of 430 nm. This assay can be used to measure DAO activity in plasma during the second and third trimester, but not in non-pregnant individuals [12].

Diamine oxidase activity was also measured in tissue extracts using hydrogen peroxide (H_2_O_2_)/horseradish peroxidase (HRP)/homovanillic acid coupling [13], but abundant antioxidants and H_2_O_2_ degrading enzymes like catalase might interfere significantly. The sensitivity of this assay using small intestinal protein extracts was comparable to the radioactive putrescine method. Schwelberger and Feurle (2007) exchanged homovanillic acid with luminol [14]. They wrote that this luminescence assay was as sensitive as the radioactive putrescine method, but they never used complex matrices like plasma or tissue extracts to compare detection limits [14]. It is likely that in particular the high serum or plasma antioxidative capacity of approximately 800 µM will significantly interfere with measuring plasma DAO activity using HRP/H_2_O_2_ coupling [15].

We recently published the development and characterization of a human DAO ELISA with limits of detection and quantification of below 1 ng/ml [7]. Nevertheless, DAO released from heparan-sulfate proteoglycans during severe mast cell activation events in mastocytosis patients reached concentrations of several hundred ng/ml, but DAO activity using the histamine degradation rate was clearly compromised compared to DAO in plasma during pregnancy [16]. Several approved medications have been described as DAO inhibitors [17]. The identification and characterization of clinically relevant DAO inhibitors will be easier if we are able to measure not only antigen concentrations but also corresponding activity.

The main limitation of current non-radioactive DAO activity assays is sensitivity and compatibility with the high antioxidant capacity of plasma. A fluorescence assay might provide the necessary increase in sensitivity. Based on the chemical structures of various fluorescent molecules such as resorufin, acridine or methylene blue we hypothesized that the triple aromatic ring generated by the condensation of delta-1-pyrroline or delta-1-piperideine with oABA is not only a chromophore but also a fluorophore.

Here, we describe the development and characterization of a novel, simple and sensitive fluorescence assay to measure DAO activity in complex matrices like plasma or tissue extracts using the natural DAO substrates cadaverine and putrescine.

## Experimental methods

### DAO activity measurements using hydrogen peroxide (H_2_O_2_)/horseradish peroxidase (HRP)/luminol coupling

**Fig. 1:**
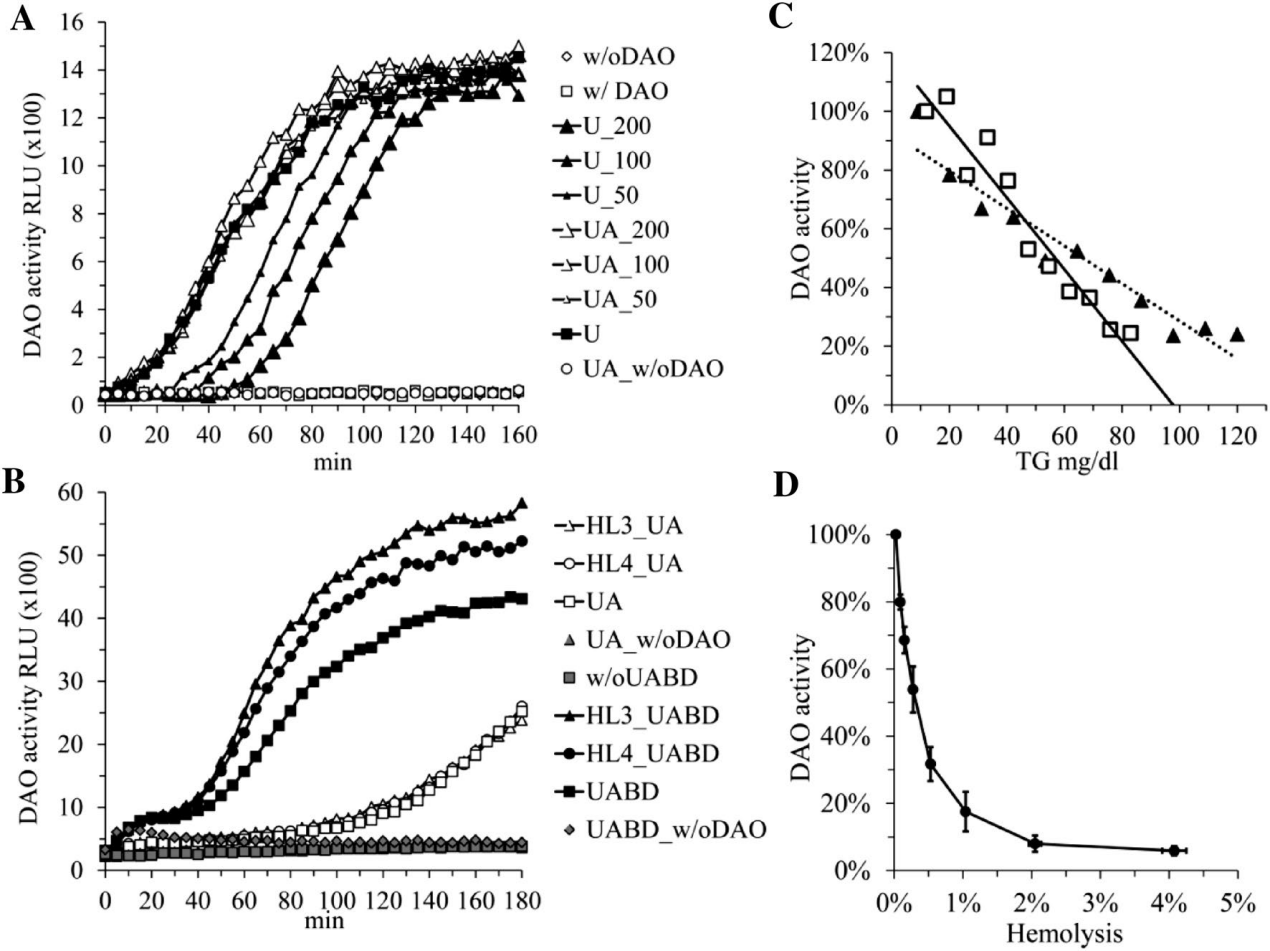
Plasma and serum DAO activity cannot be precisely measured using hydrogen peroxide (H_2_O_2_)/horseradish peroxidase (HRP)/luminol coupling. **a** Human serum with and without rhDAO spiking was left untreated or treated with 1 U/ml uricase (U) and 5 U/ml ascorbase (**a**) with or without addition of 200, 100 or 50 µM ascorbic acid; w/o DAO means without rhDAO spiking; rhDAO was spiked before addition of enzymes or ascorbic acid; **b** rhDAO activity was measured in UA treated human serum (concentrations of triglycerides [TG] 64 and cholesterol [CHOL] 157 mg/dl) without or after mixing with hyperlipidemic sera HL3 and HL4 (final concentrations TG 573, 318 and CHOL 199, 178 mg/dl, respectively). Lipids were extracted using butanol/diisopropylether (BD) as indicated; **c** Two EDTA plasma samples from two healthy volunteers were spiked with rhDAO and either treated with UA or both UA and BD. Autologous UA/non-treated and UA/BD-treated plasma was consequently mixed at various ratios obtaining triglyceride (TG) concentrations between 10 and 120 mg/dl. The DAO activities were normalized based on activity in UA/BD treated serum. The *R* values were in both regression lines > 0.96; **d** rhDAO was spiked into EDTA plasma from two donors containing different concentrations of autologous red blood cell lysate diluted in autologous plasma. All samples were treated with UA/BD. The mean (+ / − SEM) percent DAO activity determined in duplicates and the mean (+ / − SEM) percent hemolysis of the two plasma samples are plotted. In all panels DAO activity was measured using H_2_O_2_/HRP/luminol coupling and the mean relative light units (RLU) of duplicates or percentage of DAO activity normalized to the appropriate controls are shown

The DAO activity assay used in Figs. 1 and S1 is based on the luminescence assay published by [14]. We used 110 µl final volume in white luminescence plates (Porvair; Graz, Austria), which are composed of 50 µl serum or plasma treated as described below and 50 µl luminol solution from a commercial western blotting ECL kit (Amersham RPN2106; Vienna, Austria) containing 2 µg/ml horseradish peroxidase (HRP; P6782; Sigma-Aldrich; Vienna, Austria). The reaction was started with the addition of 10 µl substrate solution at a final concentration of 360 µM. The RLUs were measured in a Victor2™ 1420 Microtiter Plate Reader (Perkin Elmer; Vienna, Austria). All samples were measured in duplicate and the mean is shown. Final DAO concentrations were 0.5–2 µg/ml, quantified using a DAO ELISA which has been developed in-house [7]. We did not see relevant differences, regardless of whether DAO was added before uricase/ascorbase (UA) and butanol/diisopropylether (BD) treatment or afterwards (data not shown; see below for UA and BD description). The expression and characterization of rhDAO in Chinese hamster ovary (CHO) cells has been published [18]. In some experiments we used directly 10–20-fold concentrated CHO supernatant. There was no relevant difference in the enzyme characteristics between purified or supernatant DAO (data not shown). Pig kidney DAO (D7876), putrescine (P5780), cadaverine (C8561), histamine (53300) and ortho-aminobenzaldehyde (A9628) were purchased from Sigma-Aldrich (Vienna, Austria). Uricase (URIC-70–1701; Sekisui Enzymes; Kings Hill, UK) at 30 U/ml (30×) and ascorbase (70–6141-20 or T-53; Sekisui Enzymes; Kings Hill, UK) at 100 U/ml (40×), stocks were stored at -32 °C in single use aliquots and serum or plasma samples were pre-treated for 15 min at 37 °C. PBS was added to non-treated samples. Ascorbic acid (20×; A4544, Sigma-Aldrich; Vienna, Austria) was freshly dissolved in PBS (Gibco; Vienna, Austria). Butanol/diisopropylether lipid extraction was performed according to [19]. Briefly, 2 volumes of 1-butanol (281,549, Sigma-Aldrich; Vienna, Austria) and diisopropylether (38270, Honeywell; Vienna, Austria) at a ratio of 40–60 were added to 1 volume of serum or plasma and end-over-end rotated for 45 min. 250 µM EDTA was added to serum samples only. After extraction the mixture was centrifuged at 2000 rpm for 2 min and the aqueous (lower) phase was removed carefully. Triglyceride (TG) and cholesterol (CHOL) concentrations were reduced more than tenfold with unchanged protein concentrations [7, 19]. Triglycerides and CHOL were measured using standard methods in the clinical chemistry laboratories at the General Hospital Vienna.

### Preparation of red blood cell lysates

Whole blood anti-coagulated with EDTA was mixed with 1.5 volumes of erylysis buffer and incubated for 5 min at room temperature. Following centrifugation for 10 min at 4 °C and 350 g the supernatant was collected. This contained more than 90% of the total hemoglobin. The erylysis buffer consisted of 155 mM ammonium chloride, 100 µM EDTA and 10 mM KHCO_3_ (pH = 7.4). Autologous plasma was used to dilute the lysate. The hemoglobin concentrations were measured with the QuantiChrom kit (BioAssay Systems; QHB-Kit, Hayward; CA) or at the clinical chemistry laboratories at the General Hospital Vienna.

### Synthesis of THPQ (delta-1-pyrroline/oABA condensate) and HHPQ (delta-1-piperideine/oABA condensate)

Two µg/ml purified rhDAO, 400 µM substrates and 1 mM ortho-aminobenzaldehyde (oABA; stored at − 32 °C for 4–6 months as a 200 mM stock solution in absolute ethanol) in 20 mM Hepes at pH 7.0 containing 0.05% human serum albumin (20% Octapharma; Vienna, Austria) were incubated for 2 h at 37 °C. This oABA concentration leads to 83 mM ethanol in the samples, which does not influence DAO activity. The solutions were filtered through Amicon 3000 MWCo devices before absorption and fluorescence measurements. The condensate between delta-1-pyrroline (3,4-dihydro-2^H^-pyrrole) and oABA generates 2,3,3a,4-tetrahydro-1^H^-pyrrolo[2,1-^b^]quinazoline-10-ium (IUPAC name). This is abbreviated as THPQ. Incubation of delta-1-piperideine (2,3,4,5-tetrahydro-pyridine) and oABA generates 5,5a,6,7,8,9-hexahydropyrido[2,1-^b^]quinazoline-10-ium or HHPQ.

### Absorption and fluorescence measurements

Absorption was measured in a Synergy™ H1 Multi-Mode Microplate reader (BioTek; Winooski, Vermont, US) and scans were performed using 4 or 8 nm steps. The resolution of the Synergy™ H1 for absorbance measurements is 0.0001 optical density units. UV-compatible 96-well half-area flat bottom microplates (CLS3635 Corning® UV-Transparent Microplates; Sigma-Aldrich; Vienna, Austria) were used with a total reaction volume of 170 µl, corresponding to a light path of 1 cm.

Relative fluorescence units (RFUs) were measured with a Synergy™ H1 Multi-Mode Microplate reader using either a custom filter cube or the monochromator module. The custom filter cube is composed of an excitation filter 440/30 nm (range 425–455 nm), a dichroic mirror (DM) with a cut-off of 550 nm and an emission filter of 620/40 nm (range 600–640 nm). This filter set was selected based on the absorption maximum of the condensation product of delta-1-pyrroline with oABA at 440 nm. A filter with an excitation maximum at 460 nm (range 440–480 nm) might increase the sensitivity for measuring HHPQ, because the area under the curve (AUC) for absorption by the delta-1-piperideine/oABA fusion product (HHPQ) is increased by about 50%, whereas the control AUCs or THPQ AUCs are unchanged switching from the 440 to 460 nm filter set (data not shown). We tested such a filter and the fluorescence was increased by 43% (data not shown). Emission scans were performed using 4 or 8 nm steps with excitation at 460 nm and emission scans from 500 to 700 nm. For excitation scans we used fixed emission at 620 nm after excitation from 350 to 580 nm. For fluorescent measurements we measured a volume of 100 or 200 µl using either black or clear bottom black fluorescent plates (3915, 3615 or 3904 Corning-Costar® 96-well Black Flat Bottom Polystyrene microplates; Szabo-Scandic; Vienna, Austria).

For the detection of HHPQ (delta-1-piperideine/oABA condensate) in plasma from healthy volunteers or third trimester pregnancies we mixed 90 µl plasma adjusted to pH 7.0 with 13 µl 1 M HCl per ml of plasma with 5 µl 10% ethanol or 5 µl 20 mM oABA and 5 µl PBS or 5 µl 4 mM cadaverine. All samples were analyzed in duplicate. After 1 h incubation at 37 °C in the dark, 200 µl 7.5% TCA (99.5% trichloroacetic acid; 91228; Sigma-Aldrich; Vienna, Austria) was added and incubated for 20 min on ice. After high speed centrifugation for 10 min, 200 µl were recovered and the pH adjusted to 4.0 with 5.2 µl 10 M NaOH and 50 µl 500 mM citrate buffer pH 4.0. TCA precipitation reduced protein autofluorescence by approximately sevenfold (data not shown).

### Determination of absorption and fluorescence pH dependency using the Britton Robinson buffer

The Britton–Robinson buffer (BRB) contains a final concentration of 40 mM acetic acid, 40 mM H_3_P0_4_ and 40 mM boric acid and the pH was adjusted with NaOH from pH 2 to pH 12 [20]. Synthesized THPQ and HHPQ were diluted with the BRB buffer 13.3-fold to 30 µM and absorption and fluorescence measured. The BRB buffer contains different ionic strengths after pH adjustment. The ionic strength at pH 2 is 0.02, at pH 8 = 0.095 and increases to 0.128 at pH 12. We, therefore, also tested whether increased NaCl concentrations influence fluorescence of THPQ and HHPQ.

### HHPQ and resorufin generation in PBS containing 0.05% HSA

For the direct comparison of HHPQ and resorufin generation using different rhDAO concentrations (Fig. 5) we used the conditions discussed below. For HHPQ, 170 µl PBS with 0.05% HSA, 10 µl of different rhDAO concentrations and 10 µl 20 mM oABA were mixed. For Amplex Red oxidation, 170 µl PBS with 0.05% HSA and 1 µg/ml HRP, 10 µl of different rhDAO concentrations and 10 µl 2 mM Amplex Red were mixed. The reactions were started via the addition of 10 µl 2 mM cadaverine or PBS and incubated at 37 °C. Under these conditions direct measurements within the microplate reader are possible with low autofluorescence. Absorption of HHPQ and resorufin were measured using 460 nm and 570 nm, respectively, and fluorescence using the custom filter cube (Ex440 ± 30/DM550/Em620 ± 20) or Ex550/Em590 for resorufin using the monochromator module with 16 nm bandwidth. All samples were run in duplicate.

### HHPQ and resorufin generation in EDTA plasma

For the direct comparison of HHPQ and resorufin generation using different rhDAO concentrations in plasma (Fig. 6a, b) we used the following conditions: For HHPQ, 85 µl EDTA plasma, 5 µl of different concentrations of rhDAO and 5 µl 20 mM oABA or 5 µl 10% ethanol (oABA matrix) were mixed. For Amplex Red oxidation 85 µl EDTA plasma containing 1 µg/ml HRP, 5 µl of different concentrations of rhDAO and 5 µl 2 mM Amplex Red or 10% DMSO (Amplex Red matrix) were mixed. The reactions were started via the addition of 5 µl 2 mM cadaverine or PBS and incubated for 3 h at 37 °C in the dark. After the incubation period TCA precipitation was performed as described above. The HHPQ samples were adjusted to pH 4.0 using 50 µl of 500 mM citrate buffer and the resorufin samples to pH 7.8 using 50 µl of 500 mM potassium phosphate buffer pH 8.0. Resorufin fluorescence is reduced below pH 7.0. Absorption and fluorescence were measured as described above.

### Administration of rhDAO to rats and fluorescence-based DAO activity measurements

The experimental protocols for mice and rats were approved by the local Animal Welfare Committee and the Federal Ministry of Science, Research and Economy (Vienna; Austria) and conducted in full accordance with the ARRIVE guidelines [21]. The exact procedure of inserting a vascular access port into the Vena jugularis (Rodent Vascular Access Port with detachable Silastic Catheter, Hugo-Sachs Electronic Harvard Apparatus; Germany) and the standardized housing conditions at the Animal Care Facility at the Department for Biomedical Research, Medical University Vienna, will be described elsewhere.

Recombinant hDAO expressed and purified as described [18] was injected into approximately 400 g rats in a volume of 600 µl PBS at 1 mg/kg. At the indicated time-points 500 µl blood was drawn and anticoagulated with citrate. Sodium chloride was used to replace the blood volume. After preparation of plasma samples were stored at − 32 °C. Rat plasma was diluted 1–10 using 0.05% HSA in PBS and DAO activity measured as described above for plasma samples. Cadaverine was used as substrate and HHPQ detection was performed using the custom filter cube. Human DAO antigen concentrations were determined using a DAO ELISA [7].

### Preparation of protein extracts from DAO wild-type and knock-out mice

Diamine oxidase deficient mice were generated from frozen embryos obtained from the European Mouse Mutant Archive. Mice were kept according to the animal protocol GZ 66.009/0160-WF/V/3b/2016 which has been approved by the Austrian Agency for Health and Food Safety. Organs were harvested on ice and slices of 20–100 mg were immediately frozen in liquid nitrogen. Tissue pieces were added to 10 volumes of ice–cold 20 mM potassium phosphate buffer (pH 7.2) supplemented with 0.5% proteinase inhibitor cocktail (P8340, Sigma-Aldrich; Vienna, Austria) and homogenized at 3000 rpm for 1 min using Lysing Matrix tubes E (116914050, MP Biomedicals; Eschwege, Germany) and the Precellys24 tissue homogenizer (432–3750, VWR; Vienna, Austria). Tissue homogenates were centrifuged at 12,000 *g* at 4 °C for 20 min and protein content of the supernatants was measured using the QuantiPro BCA Assay Kit (QPBCA-1KT, Sigma Aldrich; Vienna, Austria). Protein concentrations of tissue supernatants ranged between 5 and 18 mg/ml.

85 µl of protein extracts diluted with potassium phosphate buffer to 500 µg/ml were used for the fluorescence assay as described above for plasma samples. A final concentration of 20 µM aminoguanidine (AG) was used as a potent irreversible DAO inhibitor. If other enzymes without a topaquinone in the active center are able to oxidize cadaverine, the fluorescence without substrate and with substrate and AG should be different. The hydrazine group of AG covalently binds to the topaquinone, and 20 µM AG irreversibly inhibits DAO activity by more than 95% (data not shown). If DAO is the only enzyme able to deaminate cadaverine generating delta-1-piperideine, the fluorescence between the samples without substrate and AG should be similar.

### Ethics

The study numbers for the collection of blood samples from healthy volunteers and women in their third trimester are EC:2030/2013, EC:1810/2015 and EC:1666/2012. All healthy volunteers provided their informed consent before the collection of blood samples. All procedures were performed in accordance with the ethical standards of the responsible committee on human experimentation (institutional and national) and the 1975 Declaration of Helsinki (2013 revised edition).

## Results

In 2007 Schwelberger and Feurle published that H_2_O_2_/HRP/luminol coupling is as sensitive as the state of the art DAO activity assay using radioactive putrescine [14]. Nevertheless, the performance of the luminescence assay in complex matrices such as plasma or tissue extracts has not been described. When we spiked rhDAO into human serum, no luminescence could be measured (Fig. 1a). After treatment with uricase, 50% of the peak DAO activity was present after 40 min (Fig. 1a). Different concentrations of ascorbic acid delayed the appearance of DAO activity, which could be overcome by adding ascorbase to the assay reaction mixture (Fig. 1a). Because H_2_O_2_ readily diffuses through lipid membranes [22], we hypothesized that H_2_O_2_ might diffuse into the abundant lipoprotein particles, thus escaping oxidation by HRP. Consequently, disrupting the lipid particles using butanol/diisopropylether (BD) lipid extraction should result in faster appearance of the DAO activity signal. Human serum treated with uricase (U) and/or ascorbase (A) and after lipid extraction (BD) allowed fast measurement of pig kidney DAO (Fig. S1), but also of rhDAO activity (Fig. 1b). There was a strong negative correlation between DAO activity and triglyceride concentrations (Fig. 1c). The 50% inhibitory concentration was approximately 60 mg/dl, which is present in most human plasma samples. Finally, as expected, hemolysis severely interferes with H_2_O_2_/HRP/luminol coupling with an IC50 of approximately 0.3% hemolysis or a hemoglobin concentration of 460 µg/ml assuming 150 mg/ml hemoglobin concentration in whole blood (100%) (Fig. 1d). Hemolysis might be less of an issue with human serum or plasma samples, but red blood cells from rat or mice hemolyze more easily, severely compromising H_2_O_2_/HRP/luminol coupling as method for DAO activity measurements. In addition, BD lipid extraction is not without pitfalls. First, peroxides accumulate in diisopropylether. Second, the solubility of butanol in water is 1 M or 7.7%, partially interfering with the DAO activity measurements (data not shown) and third, organic solvents are not environmentally friendly. In conclusion, an H_2_O_2_/HRP/luminol-coupled DAO activity assay is not suitable for the measurement of plasma or serum DAO oxidation capacity.

Holmsted and Tham (1959) measured DAO activity using absorption measurement of the chromophore obtained after condensation of oABA with autocyclized delta-1-pyrroline at 430 nm [11] (Fig. S2). Nevertheless, the relatively low extinction coefficient of the delta-1-pyrroline /oABA condensate (1860 M^−1^ cm^−1^) limits assay sensitivity. Human plasma absorption at 430 nm was 1.2–1.8 measuring three samples from healthy volunteers, precluding absorption measurements using minimally diluted plasma (data not shown).

**Fig. 2:**
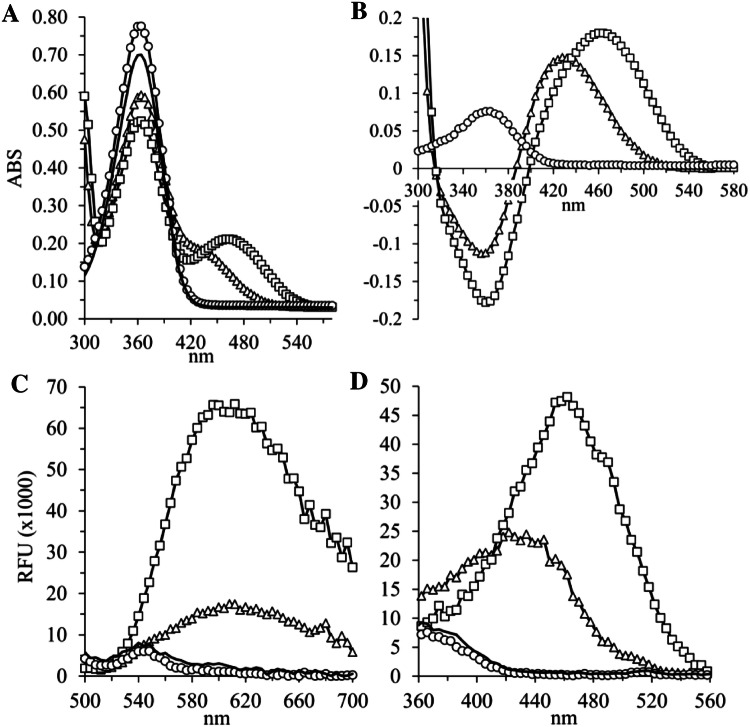
The condensates of oABA with delta-1-pyrroline (THPQ) or delta-1-piperideine (HHPQ) are not only quinazoline chromophores but also novel fluorophores. Two µg/ml purified rhDAO in 20 mM Hepes (pH 7.0) containing 0.05% human serum albumin (HSA) was incubated for 2 h at 37 °C with 1 mM ortho-aminobenzaldehyde (oABA) and 400 µM putrescine, cadaverine, histamine or Hepes buffer followed by ultrafiltration using a 3 kDa MWCO device; **a** Absorption (ABS) was measured from 300 to 700 nm after fivefold dilution using water in UV-compatible half-area plates; **b** Specific absorption curves after subtracting the Hepes buffer control data; **c** Fluorescence emission was measured between 500 and 700 nm after excitation at 460 nm; **d** Fluorescence emission was measured at 620 nm after excitation between 360 and 560 nm; White squares (□) represent cadaverine as substrate; White triangles (Δ) putrescine; White circles (○) histamine and black line (▬) no substrate. *MWCO*  molecular weight cut-off, *RFU* relative fluorescence units

Because several fluorescence compounds are linear triple aromatic ring structures, we hypothesized that the condensation product of delta-1-pyrroline (3,4-dihydro-2^H^-pyrrole) with oABA 2,3,3a,4-tetrahydro-1^H^-pyrrolo[2,1-^b^]quinazoline-10-ium (THPQ) or the condensation product of delta-1-piperideine (2,3,4,5-tetrahydro-pyridine) with oABA 5,5a,6,7,8,9-hexahydropyrido[2,1-^b^]quinazoline-10-ium (HHPQ) (Fig. S2) are not only chromophores but also fluorophores. Figure 2 shows the absorption and fluorescent profiles of freshly synthesized HHPQ and THPQ in buffer matrix at pH 7.0. Peak absorption of THPQ and HHPQ at 430 and 460 nm, respectively, coincide with the excitation maxima. The emission maxima are comparable at approximately 620 nm for both compounds. The calculated extinction coefficient for HHPQ is 2242 M^−1^ cm^−1^ compared to 1860 M^−1^ cm^−1^ for THPQ. Although the extinction coefficient of HHPQ is only increased by 17%, the fluorescence signal is consistently 200 to 250% higher. The predicted molecular weights of 173 Da for THPQ and 187 Da for HHPQ with corresponding MS2 fragments have been verified, supporting the proposed structures (Fig. S3).

We also incubated rhDAO with histamine to see whether imidazole acetaldehyde or histamine per se is undergoing condensation with oABA. It is known that histamine fuses with o-phthalaldehyde to form ill-characterized fluorophores [23–25]. Histamine or imidazole acetaldehyde likely fuses with oABA, because the oABA signal is consistently higher in the presence of histamine but there is no obvious wavelength shift and no fluorescence signal (Fig. 2b). Nevertheless, after a few days of storage histamine per se and imidazole acetaldehyde generated with rhDAO showed a specific fluorescence signal. The highest signal with imidazole acetaldehyde was obtained during storage at − 32 °C. This “histamine peculiarity” is described in the Supplement (Text and Fig. S7). A specific and sensitive assay to trap generated imidazole acetaldehyde would be interesting, because no sensitive methods have been described to measure imidazole acetaldehyde specifically. However, as we have described in the Supplement, histamine also generates a fluorescence signal and the signal with imidazole acetaldehyde at room temperature and 4 °C is at least tenfold lower compared to HHPQ, although specific over histamine at these temperatures (Fig. S7). Neither spermidine nor spermine generated a chromophore or a fluorophore after incubation with DAO and oABA, although both endogenous polyamines are substrates for DAO (data not shown).

**Fig. 3:**
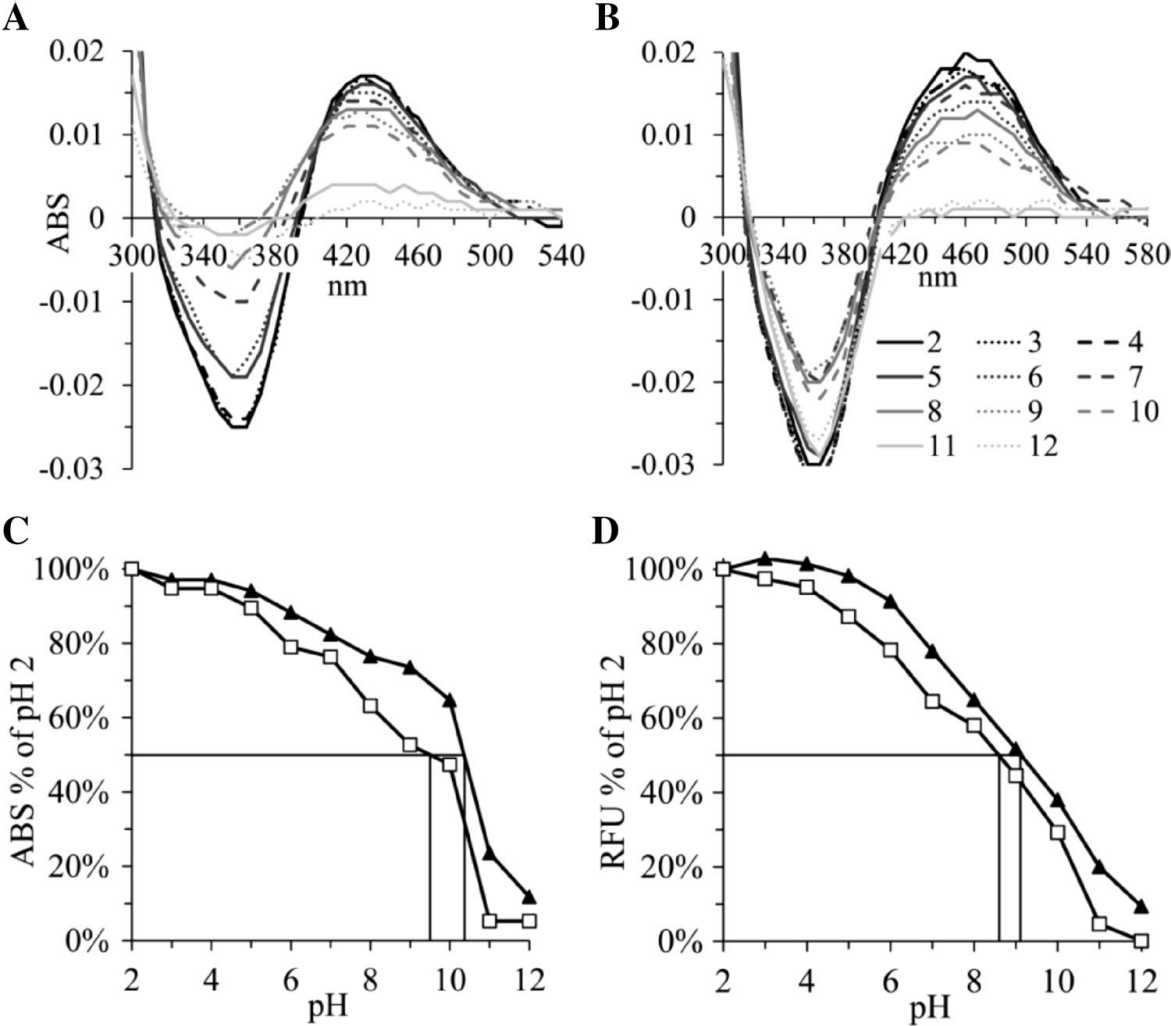
Absorption and fluorescence of THPQ and HHPQ are pH dependent with p*K*_a_ values between 8.5 and 10.5. Absorption (ABS) of freshly synthesized THPQ and HHPQ at 30 µM after 1 to 13.3 dilutions with Britton Robinson buffer at pH levels between 2 and 12 was measured from 300 and 580 nm. ABS data from Hepes buffer controls without substrate during synthesis were subtracted and the specific ABS of THPQ (**a**) and HHPQ (**b**) are presented. The numbers in Panel B below the *x*-axis represent the pH values; pH 2, 3 and 4 data are shown as black, dotted and dashed lines, respectively, and different grey shades are used for higher pH units; in **c** the ABS data from **a** at 430 nm (black triangles ▲) and **(b)** at 460 nm (white squares **□**) are normalized based on the ABS values at pH 2.0 (100%); In **d** relative fluorescence units (RFU) measured using the custom filter cube with excitation at 440 ± 15 nm, a dichroic mirror with a cut-off at 550 nm and emission at 620 ± 20 nm were normalized to the values at pH 2.0 (100%). THPQ data are shown as black triangles (▲) and HHPQ as white squares (**□**).

The Britton Robinson buffer system between pH 2 and pH 12 was used to measure the pH dependency of the absorption and fluorescence intensity of THPQ and HHPQ (Fig. 3). The pKa values for THPQ and HHPQ for absorption and fluorescence were approximately 10.5, 9.5 and 9.0, 8.5, respectively. These two fluorophores could repeatedly be excited and measured at pH values below 7 (Fig. S4A and B). Fluorescence intensity was not influenced by up to 500 mM NaCl concentration (Fig. S4C). The polar protic solvents water followed by ethanol and methanol are the preferred matrices, in contrast to the aprotic polar solvents acetone and DMSO, which severely interfered with fluorescence signal intensity measurements (Fig. S4D, E).

THPQ and HHPQ also generated new absorption peaks at 246 nm (data not shown) and 290 nm (Fig. 2a), but the signal to noise ratios after excitation at 246 nm or 290 nm are lower compared to 430/460 nm. Further characterization of the absorption and fluorescence properties of THPQ and HHPQ will be described elsewhere.

**Fig. 4:**
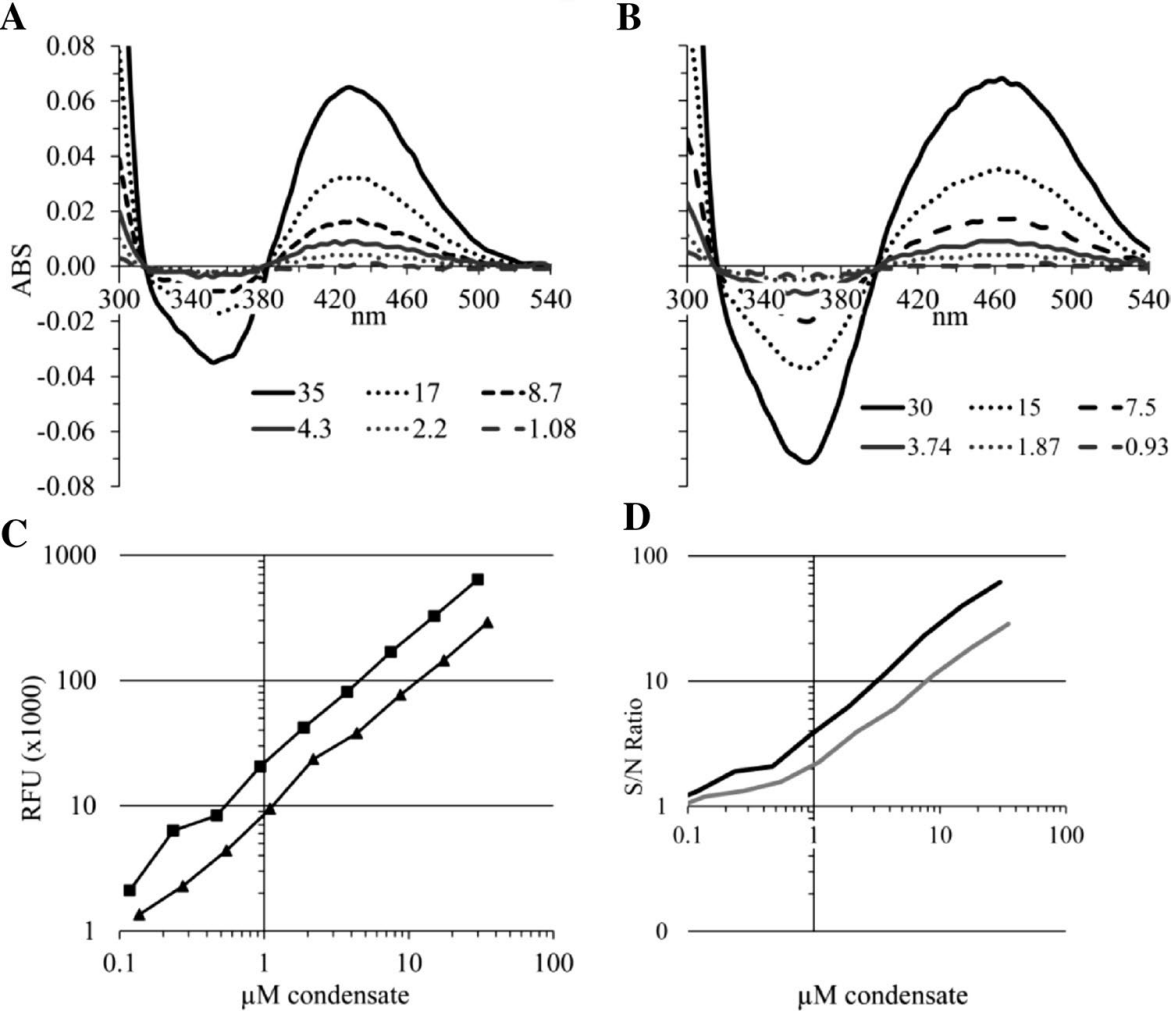
Detection limits for THPQ and HHPQ in PBS. Freshly synthesized THPQ and HHPQ were diluted with PBS to the indicated concentrations shown in **a** for THPQ and **b** for HHPQ and absorption (ABS) measured between 300 and 540 nm. The THPQ and HHPQ concentrations were calculated based on the extinction coefficients of 1860 M^−1^ cm^−1^ for THPQ and 2242 M^−1^ cm^−1^ for HHPQ; The PBS control containing oABA was subtracted, and therefore, specific ABS curves are shown. **c** Fluorescence (RFU = relative fluorescence units) of the same samples was measured using the custom filter cube with excitation at 440 ± 15 nm, a dichroic mirror with a cut-off at 550 nm and emission at 620 ± 20 nm and plotted against the THPQ (black triangles ▲) and HHPQ (black squares ■) concentrations; The slopes for THPQ and HHPQ are 8280 and 21,390, respectively; **d** The signal over noise ratios were calculated for the fluorescence data shown in **c**. THPQ = grey; HHPQ = black lines; S/N = Signal/Noise

In the next experiments we determined the detection limits in a simple buffer matrix. Freshly synthesized THPQ and HHPQ were diluted in PBS followed by absorption and fluorescence measurements (Fig. 4). The detection sensitivity for absorption measurements is about the same for THPQ and HHPQ at approximately 2–4 µM. Fluorescent-based sensitivity limits are approximately 0.3 µM for THPQ and 0.1 µM for HHPQ, which is 20–40-fold lower compared to absorption measurements. Using fluorescence measurements in clean buffer matrices 1 µM THPQ and HHPQ consistently generate approximately 8000 and 21,000 RFUs (Fig. 4c and data not shown).

**Fig. 5:**
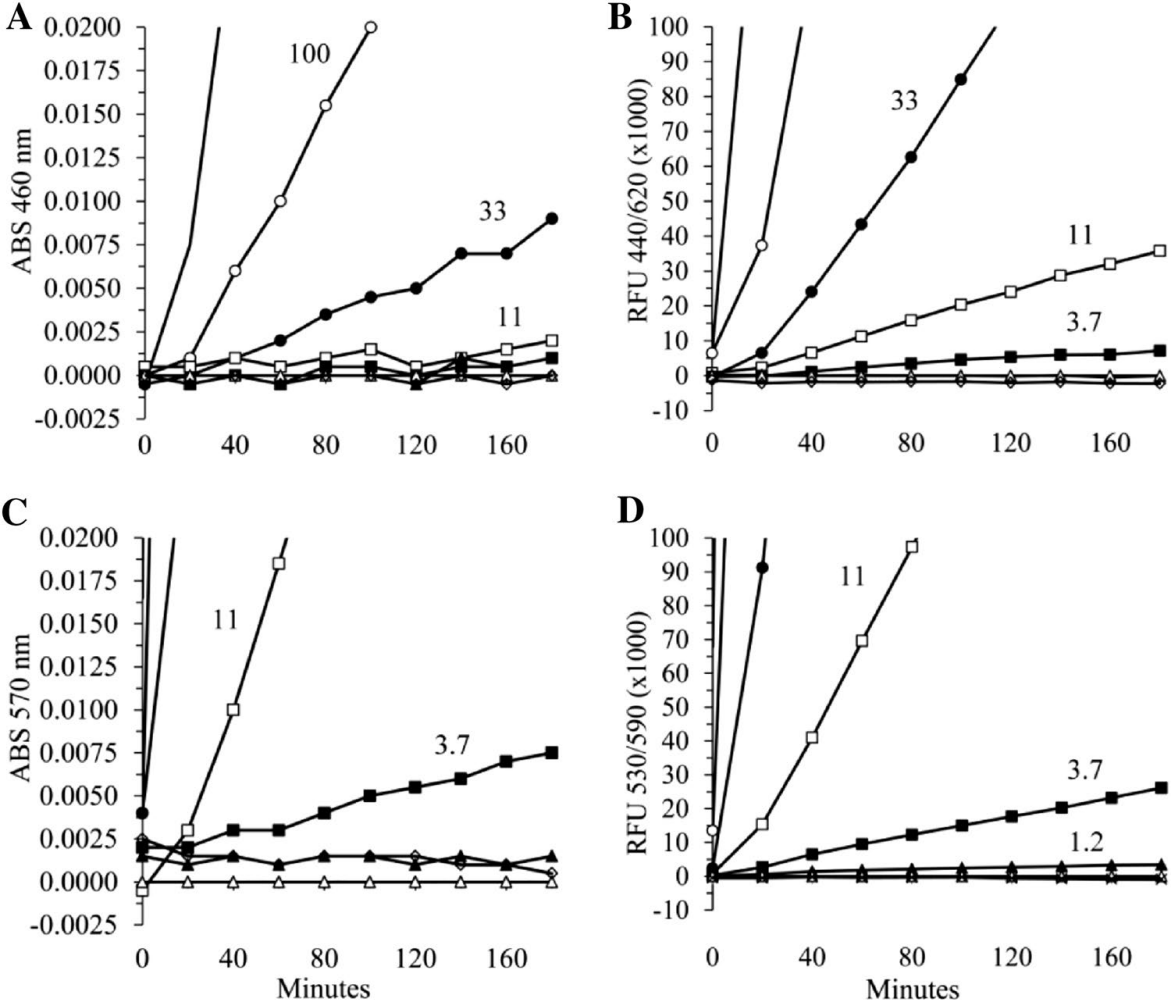
Fluorescence measurements of DAO activity using HHPQ detection in comparison to hydrogen peroxide (H_2_O_2_)/horseradish peroxidase (HRP)/Amplex Red coupling. **a** Six rhDAO concentrations were incubated with 1 mM oABA and 100 µM cadaverine in PBS (pH 7.4 containing 0.05% human serum albumin) at 37 °C and absorption (ABS) measured at 460 nm every 20 min for 3 h; **b** Fluorescence (RFU = relative fluorescence units) of the same samples was measured using the custom filter cube with excitation at 440 + / − 15 nm, a dichroic mirror with a cut-off at 550 nm and emission at 620 + / − 20 nm; **c** The same samples described in **a** were incubated not with oABA but with 1 µg/ml HRP and 100 µM Amplex Red and ABS was measured at 570 nm; **d** The RFUs of the samples shown in Panel C were determined using excitation at 530 nm and emission at 590 nm. All samples were run in duplicate and the means are shown. The following symbols represent 300 (▬); 100 (○); 33 (●); 11 (□); 3.7 (■); 1.23 (▲) and 0 (Δ) ng/ml rhDAO and are partially shown in the panels; the white diamonds (◊) represent no DAO and no cadaverine. The ABS data have been normalized to the data using 0 ng/ml rhDAO

In the next experiment we determined the minimum concentration of rhDAO detectable with HHPQ versus Amplex Red using absorption and fluorescence measurements in PBS pH 7.4 with 0.05% human serum albumin (Fig. 5). The fluorescence-based analysis of HHPQ is at least 10-times more sensitive compared to absorption measurements (Fig. 5b versus 5a). Measurements of the fluorescence of resorufin are able to detect 1.2 ng/ml DAO and are more sensitive compared to using HHPQ detection, but by no more than threefold. Nevertheless, resorufin was detected using the monochromator with 16 nm bandwidth. For the excitation and emission of HHPQ we used a custom filter cube with 30 and 40 nm bandwidths. The ratio of the extinction coefficients between HHPQ (2242 M^−1^ cm^−1^) and resorufin (54,000 M^−1^ cm^−1^) is 24. The squared correlation coefficients of the slopes of the regression lines plotting rhDAO in ng/ml versus absorption or fluorescence units of HHPQ or resorufin is > 0.99 in all 4 cases, indicating excellent linearity and rhDAO concentration dependency.

**Fig. 6:**
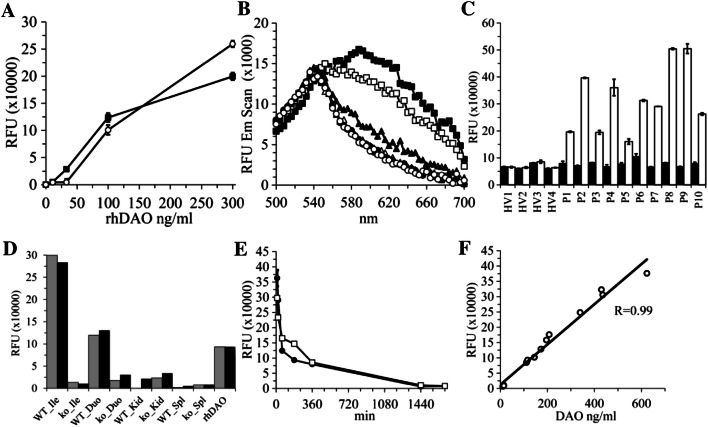
The HHPQ fluorophore can be used to measure endogenous and exogenous DAO activity in complex matrices like human and rat plasma or mouse tissue extracts. **a** Indicated rhDAO concentrations were incubated in EDTA plasma with 200 µM cadaverine (CAD) for 3 h at 37 °C. The black square (■) series contained 1 mM oABA and the parallel-prepared white circles (○) 100 µM Amplex Red and 1 µg/ml horseradish peroxidase. After trichloroacetic acid (TCA) protein precipitation the pH was adjusted to 4.0 for measuring HHPQ and to 7.8 for measuring resorufin. Relative fluorescence units (RFUs) were determined using the custom filter for HHPQ and the monochromator using excitation at 550 nm and emission at 590 nm for resorufin. The mean of duplicates (+ / − SEM) are presented; **b** Emission scans of HHPQ after excitation at 460 nm. The following symbols represent 300 (■), 100 (□), 33 (▲), 11 (Δ), 0 (●) ng/ml rhDAO; White circles (○) show fluorescence without DAO and without oABA. **c** Four EDTA plasma samples from healthy volunteers (HV1–HV4) and 10 EDTA plasma samples from 3rd trimester pregnancies were incubated with 1 mM oABA and either PBS (black columns) or 200 µM CAD (white columns) for 1 h at 37 °C. After TCA precipitation and pH adjustment to 4.0, RFUs were measured using the custom filter cube. The means of duplicates (+ / − SEM) are shown. **d** Protein extracts of different organs from wild-type and DAO knock-out mice at 500 µg/ml were incubated with 1 mM oABA and either water, 400 µM CAD or 400 µM CAD plus 20 µM aminoguanidine (AG); RFUs were measured using the custom filter cube after 60 min at 37 °C. Black and grey columns represent the specific RFUs after subtracting the RFUs from the incubations without CAD or with CAD plus AG, respectively. *Ile* Ileum, *Duo* Duodenum, *Kid* Kidney, *Spl* Spleen, rhDAO at 500 ng/ml, *WT* wildtype mice; *ko* DAO knockout mice; **e** 1 mg/kg rhDAO was intravenously injected into two rats and plasma collected at different time points. Plasma samples were tenfold diluted and incubated with 1 mM oABA and 200 µM CAD for 90 min at 37 °C. RFUs were measured after TCA precipitation. The means (+ / − SEM) of duplicates are shown. **f** Correlation between RFUs (Panel **e**) and DAO antigen concentrations measured using our human DAO ELISA

Before measuring HHPQ and resorufin in plasma we added 1 mM oABA final concentration to 5 plasma samples from healthy volunteers and measured absorbance and fluorescence with the custom filter cube after trichloroacetic acid (TCA) precipitation (Fig. S5). Addition of oABA to the 5 plasma samples generated an absorption signal at 460 nm of 104% (SD = 6.0%) compared to the samples without oABA. The increase in fluorescence of 11% (SD = 2.9%) corresponds to approximately 2–3 µM HHPQ. This signal might be caused by circulating delta-1-pyrroline-5-carboxylate and delta-1-piperideine-6-carboxylate. The fluorescence signal of HHPQ and of resorufin in plasma under comparable conditions shows that HHPQ detection is more sensitive compared to H_2_O_2_/HRP/Amplex Red coupling (Fig. 6a). Below 40 ng/ml rhDAO Amplex Red conversion seems totally inhibited. The fluorescence signals at 100 ng/ml rhDAO are comparable. The flattening HHPQ signal at 300 ng/ml DAO might already indicate substrate consumption. The emission scan showed the expected fluorescence between 600 and 640 nm (Fig. 6b). Diamine oxidase in plasma of pregnant women can be readily measured after only 1 h incubation at 37 °C (Fig. 6c), and the excitation and emission scans show the expected curves only after addition of cadaverine (Fig. S6). Protein extracts from DAO knockout mice showed strongly reduced fluorescence in the ileum and duodenum but not the kidney and spleen (Fig. 6d), with the latter two organs showing no or minimal DAO expression in mice (GEO accession number GDS3142; [26]). After intravenous administration of 1 mg/kg rhDAO, circulating rhDAO can be readily measured in rat citrate plasma (Fig. 6e) with a very high correlation between measured fluorescence and human DAO ELISA concentrations (Fig. 6f).

From the in vivo rat rhDAO administration experiment (Fig. 6e) we diluted plasma samples ten-fold and, therefore, could directly measure fluorescence at 0, 30, 60 and 90 min. In one rat at three times points (10, 20 and 60 min after administration of rhDAO) the 200 µM CAD substrate was almost consumed at 60 min (data not shown). The mean (SEM) RFU increase from 60 to 90 min of the 10, 20 and 60 min samples was only 13% (3.5%). Therefore, we assumed that after 60 min 87% of the 200 µM CAD or 174 µM were consumed. The mean (SEM) DAO antigen concentration circulating 10, 20 and 60 min after administration was 862 (67) ng/ml. Therefore, enzyme activity was 3.4 µmol/min/mg DAO. In the same assay we used rhDAO as control in PBS with 0.05% HSA and using 1 µg/ml DAO the RFUs from 60 to 90 min increased by only 7% indicating substrate consumption (data not shown). Therefore, we assumed that after 60 min 93% or 186 µM of CAD were consumed. This converts to 3.1 µmol/min/mg DAO. These values are close to our published values with rhDAO from CHO cells of 3.2 using absorption measurements of THPQ or to Elmore et al. (2002) of 2.9 µmol/min/mg with insect cell-derived rhDAO using H_2_O_2_/HRP coupling [1, 18]. All calculations were performed using V_max_ conditions with no corrections for the minimal *K*_cat_ differences between PUT and CAD [1]. Conversion calculations with underlying assumptions have been published [7]. The conversion of several published activity measurements into ng/ml are close to the DAO ELISA measured antigen concentrations [7].

Southren (1964) measured DAO activity in 15 healthy women at parturition employing the radioactive ^14^C-putrescine assay with 95% plasma [39]. Using the average plateau activity of approximately 420 units and the unit definition of 0.45 pmol/ml/min at 37 °C the DAO activity would be 189 pmol/ml/min. In the 10 pregnancy plasma samples at parturition we measured a mean DAO activity of 191 pmol/ml/min using 21,000 specific RFUs per µM substrate consumption (Figs. 4c, 6c). Cadaverine was unlikely consumed after 60 min, because the mean DAO concentration in the pregnancy samples was 122 ng/ml.

## Discussion

Although DAO celebrated already its 90th birthday, the physiological role of this enzyme is unclear [27]. Large animal data in pigs and sheep support the role of DAO in the protection from exogenous histamine [28, 29], but conclusive human data showing that increased exogenous histamine sensitivity is strongly associated with lower DAO activity are not available. The role of DAO in the degradation of endogenously-released histamine, for example during anaphylaxis or mast cell activation, is unknown. The strong increase in DAO activity and antigen concentrations during pregnancy in animals and humans does not have an ascribed function, and might be simply an epiphenomenon of the excessive expression and secretion of hDAO from fetal extravillous trophoblasts [4, 30].

Despite the lack of a clear understanding of the physiological and pathophysiological roles of DAO, a partially purified porcine kidney DAO was used for the treatment of different allergic conditions soon after its discovery [31]. Efficacy data or data showing any clinically relevant benefit using this preparation have not been published. Limited efficacy could be due to the rapid clearance from the circulation or the presence of an endogenous DAO inhibitor. Several decades ago only DAO activity assays were available but this might not have been sufficient. Antigen concentrations could not be measured. Pharmacokinetic data about this DAO formulation have not been published.

To better understand any functional role of hDAO adequate tools are necessary to measure not only antigen concentration but also activity. Nevertheless, the use of radioactive substrates is discouraged in today’s laboratory environment for multiple reasons and HRP/H_2_O_2_-coupling might be as sensitive as the radioactive putrescine assay [5], but HRP/H_2_O_2_-generated radicals generated as correlate for DAO activity might be avidly trapped by the high concentrations of antioxidants like uric acid, Vitamin C or Vitamin E or H_2_O_2_ degraded by enzymes in complex matrices like plasma or tissue extracts. In Fig. 1 we use plasma to provide strong evidence that antioxidant activity severely compromises activity data using HRP/H_2_O_2_-coupling. We were actively searching for an alternative assay with increased sensitivity compared to absorption measurements described by Holmstedt and Tham [11] and McEwen [12] but independent of H_2_O_2_.

**Fig. 7:**
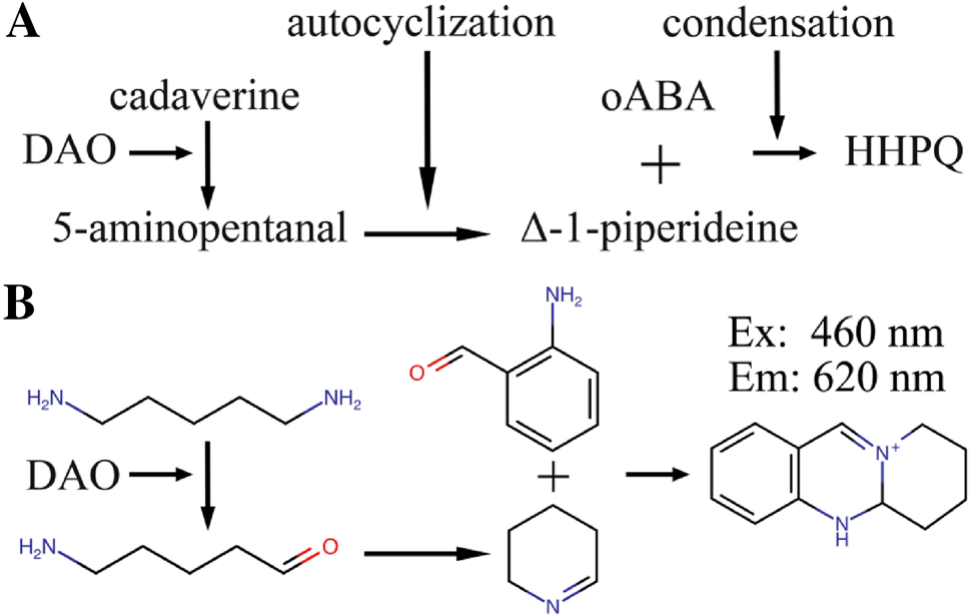
Scheme for the generation of the quinazoline fluorophore HHPQ from cadaverine via DAO oxidation. **a** After DAO oxidation of cadaverine (pentane-1,5-diamine) the released aldehyde 5-aminopentanal autocyclizes to delta-1-piperideine (2,3,4,5-tetrahydro-pyridine), which spontaneously condensates with ortho-aminobenzaldehyde (oABA) generating HHPQ or 5,5a,6,7,8,9-hexahydropyrido[2,1-^b^]quinazoline-10-ium. The release of H_2_O_2_ and NH_3_ during deamination, the release of H_2_O during autocyclization and OH^−^ after condensation and rearrangement are not shown; **b** Chemical structures; *Ex* Excitation, *Em* Emission; For the scheme using putrescine as substrate see Supplement

We confirm the proposed structures of the condensate between oABA and delta-1-pyrroline or delta-1-piperideine using mass spectrometry and show that THPQ and HHPQ are not only chromophores but also fluorophores increasing the sensitivity of the Holmstedt assay by at least tenfold. The general scheme of this assay using cadaverine as substrate including chemical structures is presented in Fig. 7.

The cadaverine-generated HHPQ consistently shows 250% higher fluorescence signal intensities compared to THPQ, which might be explained by the 6-ring structure compared to the delta-1-pyrroline-generated THPQ. At the moment we have neither data nor a conclusive hypothesis regarding the relatively large Stoke shifts of 5610 cm^−1^ (160 nm) and 6598 cm^−1^ (180 nm) for HHPQ and THPQ, respectively. Median Stoke shifts of standard fluorescent dyes are 659 cm^−1^ (24 nm; *n* = 31).

Using tissue extracts of wild-type and DAO knock-out mice we were able to show that the fluorescence signals with activity-blocked DAO and the signal without addition of cadaverine as substrate were very similar. This indicates that no other enzymes can efficiently oxidize cadaverine and generate delta-1-piperideine. The endogenous polyamine putrescine is likely diluted too highly during tissue homogenization and protein extract preparation to cause significant fluorescence after condensation with oABA. Spermine and spermidine do not generate a fluorescence signal in this assay.

The background signal in the various tissue extracts might be caused by the presence of delta-1-pyrroline-5-carboxylate and delta-1-piperideine-6-carboxylate. These Schiff bases generated within proline and lysine metabolic pathways condensate with oABA and might contribute to the background signal [32–34]. Nevertheless, under normal conditions the concentrations of both metabolites are likely low, because they are converted by reductases and dehydrogenases to metabolites unable to condensate with oABA. Individuals with very rare enzymatic defects like antiquitin deficiency or hyperprolinemia II show increased oABA condensate-specific absorption in urine [35–37] and likely also in tissue extracts, but we are not aware of published data using tissue samples.

At low pH reduced NADH but not NAD + condensates with oABA [38]. A quinazoline derivative similar to HHPQ was likely generated with an absorption peak of approximately 460 nm. There are no publications regarding whether the NADH/oABA condensates also fluoresce. These condensates might be generated after 5% TCA precipitation (pH below 2) in tissue extracts or plasma. Except for really strong DAO substrate-independent fluorescence the background signal is not a real concern, because the assay should always be performed with and without addition of cadaverine and possibly aminoguanidine.

This new fluorescent assay allows the sensitive detection of DAO activity without the use of radioactive substrates and irrespective of the antioxidant capacity of tissue extracts or plasma. It is also simple to perform and directly measures the autocyclization products of natural substrates. This method could be also adjusted to quantify submicromolar concentrations of putrescine or cadaverine generated by ornithine or lysine decarboxylases. The enzymatic activity of polyamine oxidase generating putrescine from spermidine or acetyl-spermidine might be also measured. Derivatives of delta-pyrroline like delta-1-pyrroline-2-carboxylate or similarly delta-1-piperideine-2-carboxylate might also fuse with oABA allowing fluorescent quantification with this assay. Liquid chromatographic separation of samples followed by fluorescence detection of THPQ or HHPQ likely increases the sensitivity several-fold, but also causes some of the simplicity of the assay to be lost.

In conclusion, we are now able to measure human DAO antigen concentrations with the corresponding activity and should be able to detect circumstances under which the DAO antigen concentration is high but the activity is low. This is important for investigations of possible DAO inhibitors among administered drugs. This assay is also essential to elucidate the mechanism behind the recently published data during severe mast cell activation events, where DAO activity was severely compromised despite high antigen concentrations [37]. We need to better understand how DAO activity can be inhibited, preferably before rhDAO is used for the treatment of conditions with excess circulating histamine such as anaphylaxis or mast cell activation syndrome.

## Electronic supplementary material

Below is the link to the electronic supplementary material.

Supplementary file1 (PDF 1087 kb)
