# Supplementary material for: Simple, sensitive and specific quantification of diamine oxidase activity in complex matrices using newly discovered fluorophores derived from natural substrates
Source: Inflamm Res. 2020 Jun 1;69(9):937–50. doi: 10.1007/s00011-020-01359-5 (PMC7394931; doi:10.1007/s00011-020-01359-5)
Supplement: Supplementary file 1 — Supplementary file1 (PDF 1087 kb) [file 11_2020_1359_MOESM1_ESM.pdf]

Supplement to:

Simple, sensitive and specific quantification of diamine oxidase activity in complex matrices using newly discovered fluorophores derived from natural substrates

Thomas Boehm[1], Matthias Karer[1], Elisabeth Gludovacz[1,2], Karin Petroczi[1], Marlene Resch[1], Kornelia Schuetzenberger[3], Kristaps Klavins[4], Nicole Borth[2], Bernd Jilma[1]

[1]Department of Clinical Pharmacology, Medical University of Vienna, Waehringer Guertel 18-20, 1090 Vienna, Austria

[2]Department of Biotechnology, University of Natural Resources and Life Sciences, Muthgasse 18, 1190 Vienna, Austria

[3]Center for Medical Physics and Biomedical Engineering, Medical University of Vienna, Waehringer Guertel 18-20, 1090 Vienna, Austria

[4]CeMM Research Centre for Molecular Medicine of the Austrian Academy of Sciences, Lazarettgasse 14, 1090 Vienna, Austria

Correspondence

Thomas Boehm

Department of Clinical Pharmacology, Medical University of Vienna

Waehringer Guertel 18-20, 1090 Vienna, Austria

Tel.: +43-1-40400-49580, Fax: +43-1-40400-29980

Email: thomas.boehm@meduniwien.ac.at

Supplementary Methods

Electrospray ionization mass spectrometry (ESI-MS)

The triple aromatic ring structure of the condensate between oABA and delta-1-pyrroline was proposed in 1936, but was never analyzed using NMR or mass spectrometry [1]. Freshly synthesized THPQ and HHPQ samples were analyzed using direct infusion mass spectrometry (MS). A Xevo TQ-MS mass spectrometer equipped with electrospray ionization source (ESI) was used for the analysis. A Hamilton syringe pump was used to introduce samples into the mass spectrometer at a flow rate of 10 µl per minute. The mass spectrometer settings were as follows: positive ionization mode; source temperature: 150 °C; capillary voltages: 3.5 kV; desolvation temperature: 200 °C; cone voltage: 20 V. The mass spectra were acquired between 80 and 400 m/z with a scan speed of 1 second and an acquisition time of 30 seconds. Collision-induced dissociation using argon as the collision gas and a collision energy of 30 arbitrary units was used to obtain MS2 spectra. Raw data files were used for preparing the figures in Excel. Chemicals were drawn with MarvinSketch.

Supplementary Results

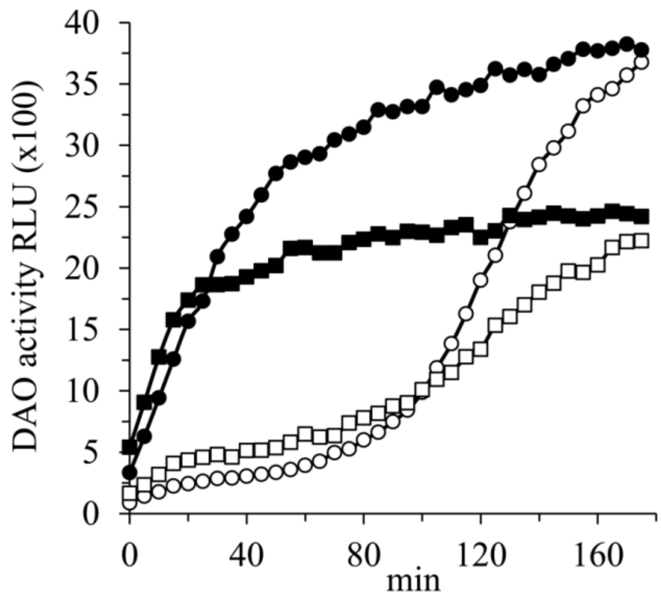

Fig. S1: Uricase/ascorbase treated serum from a healthy volunteer was spiked with 2 and 0.67 mg/ml pig kidney DAO extract and DAO activity measured without (white circles ○ 2 and white squares □ 0.67 mg/ml) or after lipid extraction using butanol/diisopropylether (black circles ● 2 and black squares ■ 0.67 mg/ml). DAO activity appears faster after lipoprotein particle disruption and lipid extraction. Based on rhDAO activity measurements the DAO concentration in the pig kidney extracts are less than 1% of the total protein concentration (data not shown).

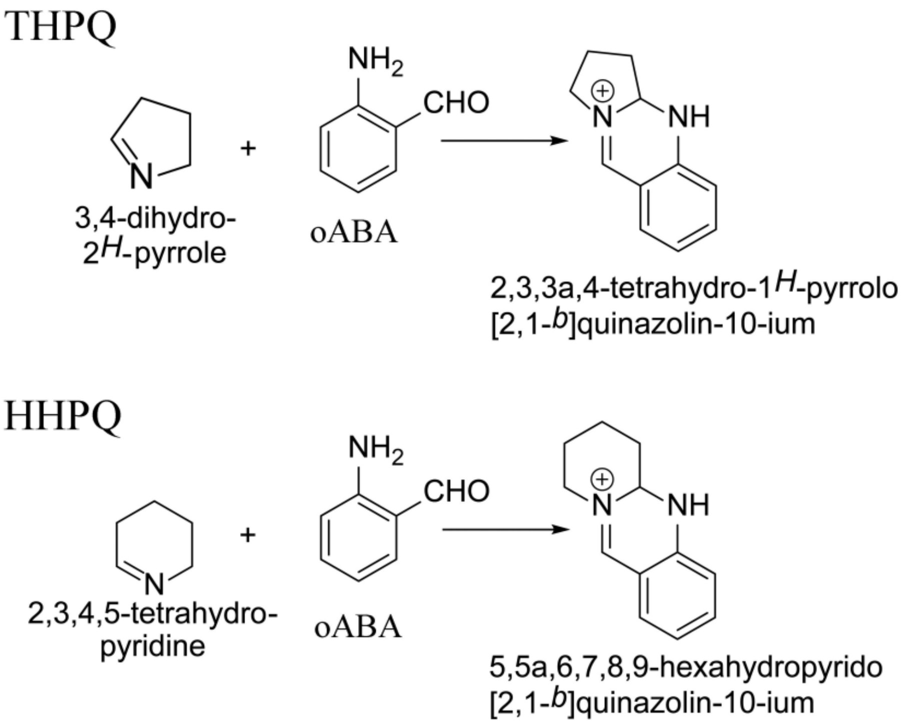

Fig. S2: Nomenclature and proposed structures of the quinazoline fluorophores THPQ and HHPQ

(a) Incubation of delta-1-pyrroline (3,4-dihydro-2^{H}-pyrrole) with ortho-aminobenzaldehyde (oABA) generates THPQ or 2,3,3a,4-tetrahydro-1^{H}-pyrrolo[2,1-^{b}]quinazoline-10-ium; **(b)** Incubation of delta-1-piperideine (2,3,4,5-tetrahydro-pyridine) with oABA generates HHPQ or 5,5a,6,7,8,9-hexahydropyrido[2,1-^{b}]quinazoline-10-ium.

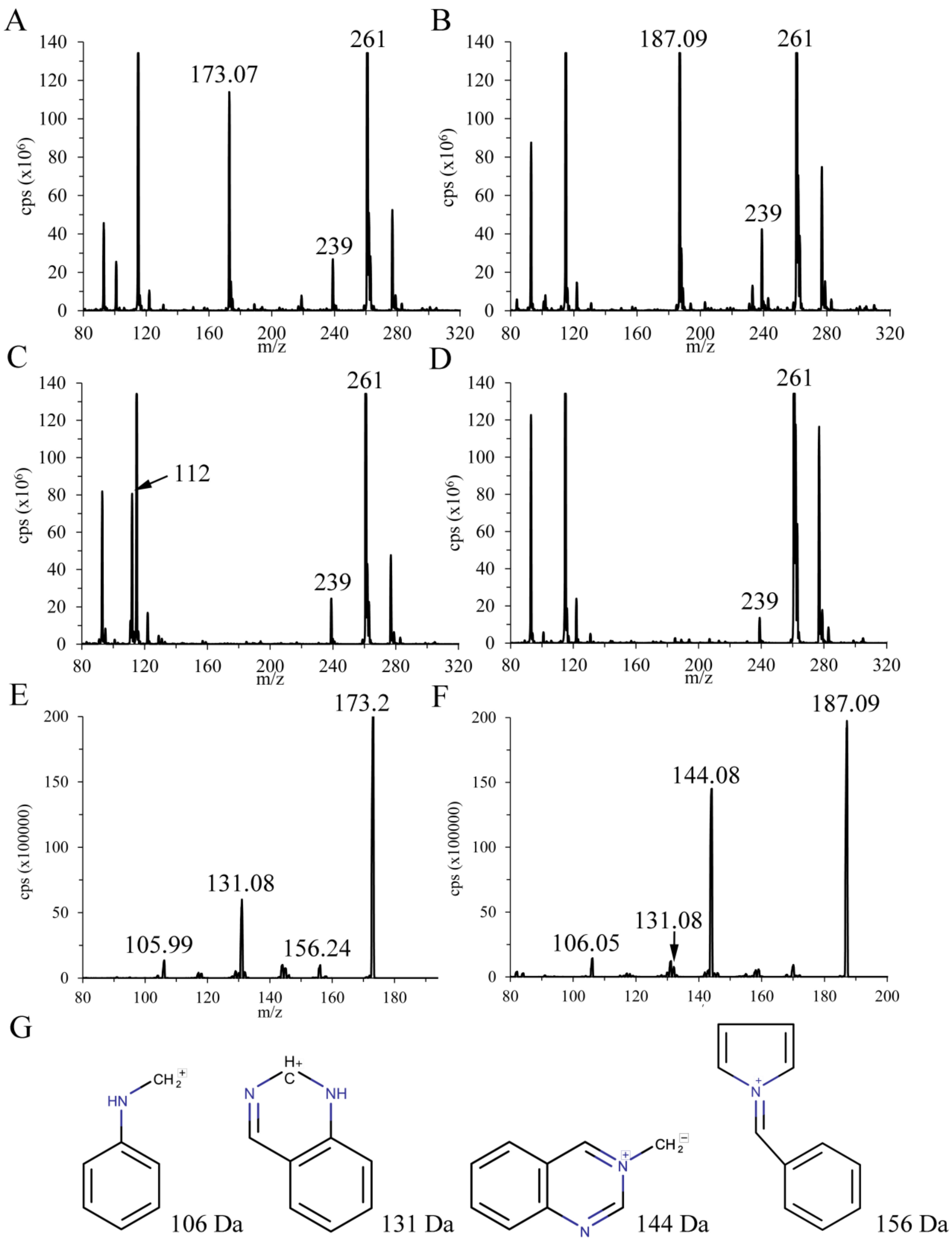

Fig. S3: Determination of the molecular weights of THPQ and HHPQ using ESI-MS

Two µg/ml rhDAO were incubated for 2 h at 37°C with 1 mM oABA and 400 µM putrescine **(a)**, cadaverine **(b)**, histamine **(c)** or Hepes buffer **(d)**. Before ESI-MS analysis samples were ultra-filtrated using a 3k Da MWCO device; Expected molecular weights for THPQ and HHPQ are 173 and 187 Da respectively; The peaks at 239.16 and 260.87 Da are likely caused by Hepes and its sodium adduct; Histamine at 112 Da is seen in Panel C. **(e)** MS/MS of the 173 Da peak with expected fragments of 156.24, 131.08 and 105.99 Da. **(f)** MS/MS of the 187 Da peak with expected fragments of 144.08, 131.08 and 106.05 Da. (**g**) MS/MS fragments.

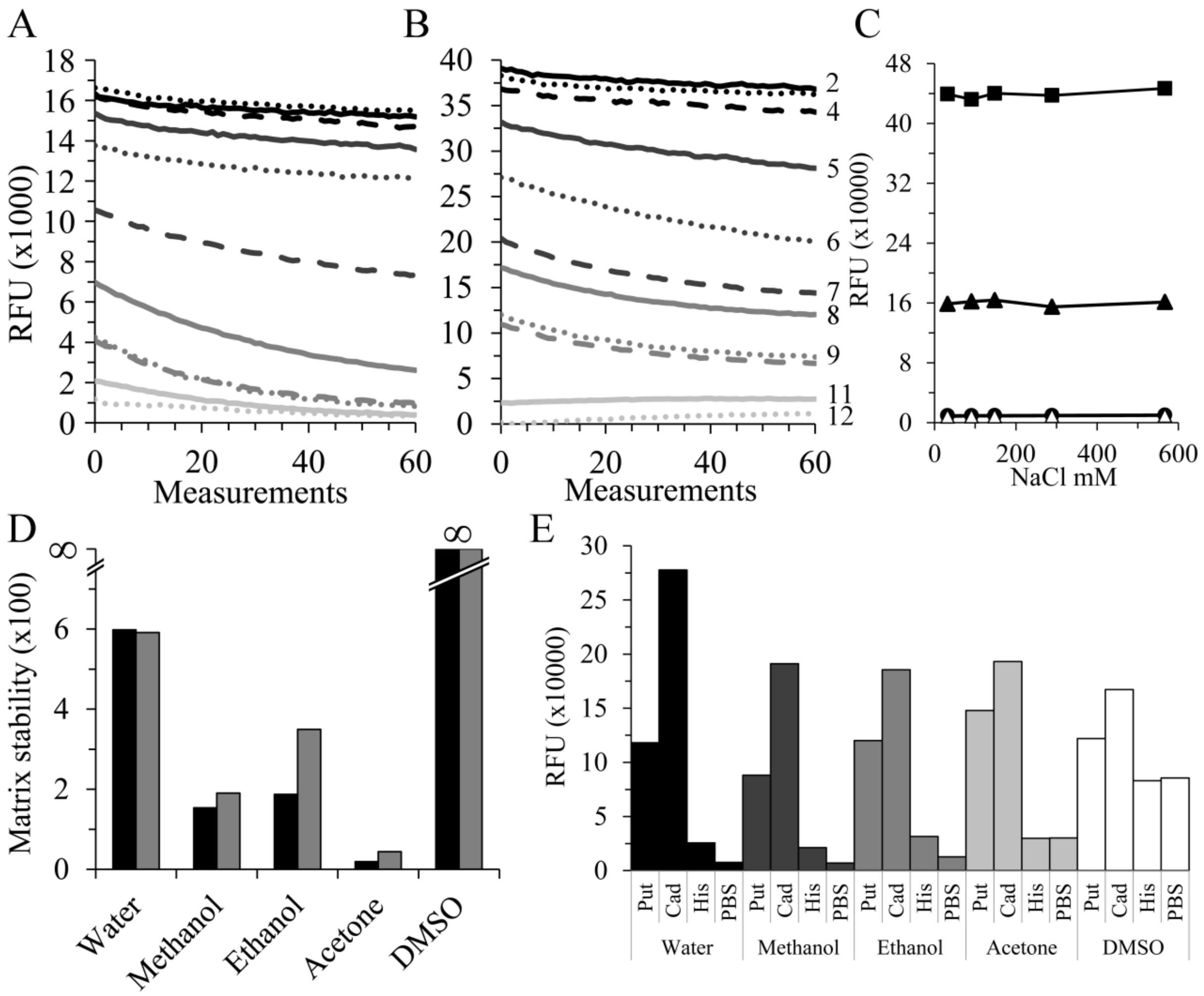

Fig. S4: THPQ and HHPQ pH stability and optimal matrix for fluorescence measurements

Thirty µM of THPQ **(a)** and HHPQ **(b)** at different pH values using the Britton Robinson buffer as indicated were measured every minute for 60 minutes using the custom filter cube. In **(c)** fluorescence of 30 µM THPQ (▲), HHPQ (■), histamine (●), or Hepes buffer (Δ) was measured at different NaCl concentrations; **(d)** HHPQ (black columns) and THPQ (grey columns) were diluted to reach a 97.5% solvent concentration and 60-times measured every minute for 60 minutes. Matrix stability was calculated from the slopes of the RFU signal over time and indicates how many measurements are necessary to reduce the RFU signal by 50%. The RFUs of THPQ and HHPQ did not decrease in DMSO (∞ “infinite” stability), but the specific signal was strongly reduced (see Panel E); both fluorophores are not stable in acetone and less than 40 measurements reduce the signal by 50%. In water extrapolated 600 measurements with the filter cube reduce the signal by 50%. **(e)** Fluorescence intensity after mixing THPQ (Put), HHPQ (Cad), histamine (His) or PBS buffer samples in different solvents. The solvent concentration was 90%. Aqueous solutions are clearly the preferred solvent.

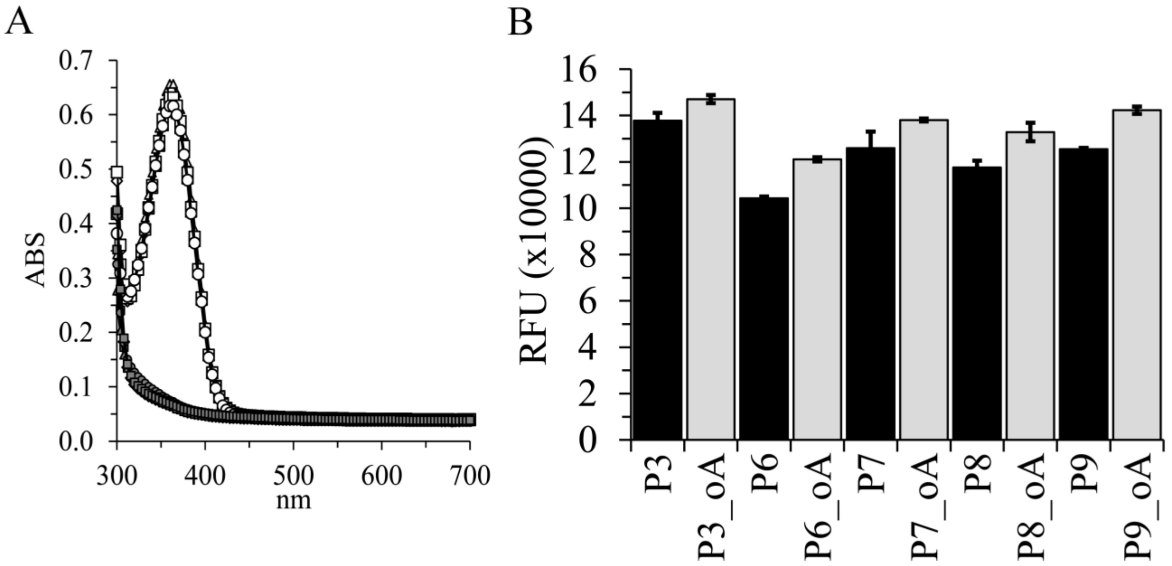

Fig. S5: Addition of 1 mM ortho-aminobenzaldehyde (oABA) only minimally increases absorption and fluorescence of plasma samples

Five different EDTA plasma samples from heatlhy volunteers were incubated in duplicates with 10% ethanol (oABA matrix) or 1 mM oABA and 400 µM cadaverine for 1 h at 37°C. After trichloroacetic acid (TCA) protein precipitation absorption (ABS, **a**) and fluorescence (RFU, **b**) was measured at pH 4.0; **(a)** White symbols with oABA and grey symbols without oABA; The mean (SD) absorbance (mean absorption at 456, 460 and 464 nm) was 104% (6.0%) with oABA compared to without oABA; **(b)** Black columns without oABA and grey with oABA; The mean (SD) RFU signal using the custom filter cube was 111% (2.9%) with oABA compared to without oABA; The increase in fluorescence adding oABA is likely caused by circulating putrescine, endogenous delta-1-pyrroline-5-carboxylate (proline metabolism) or delta-1-piperideine-6-carboxylate (lysine metabolism) and corresponds to about 2-3 µM based on the RFU signal using HHPQ; oA = ortho-aminobenzaldehyde; P = plasma.

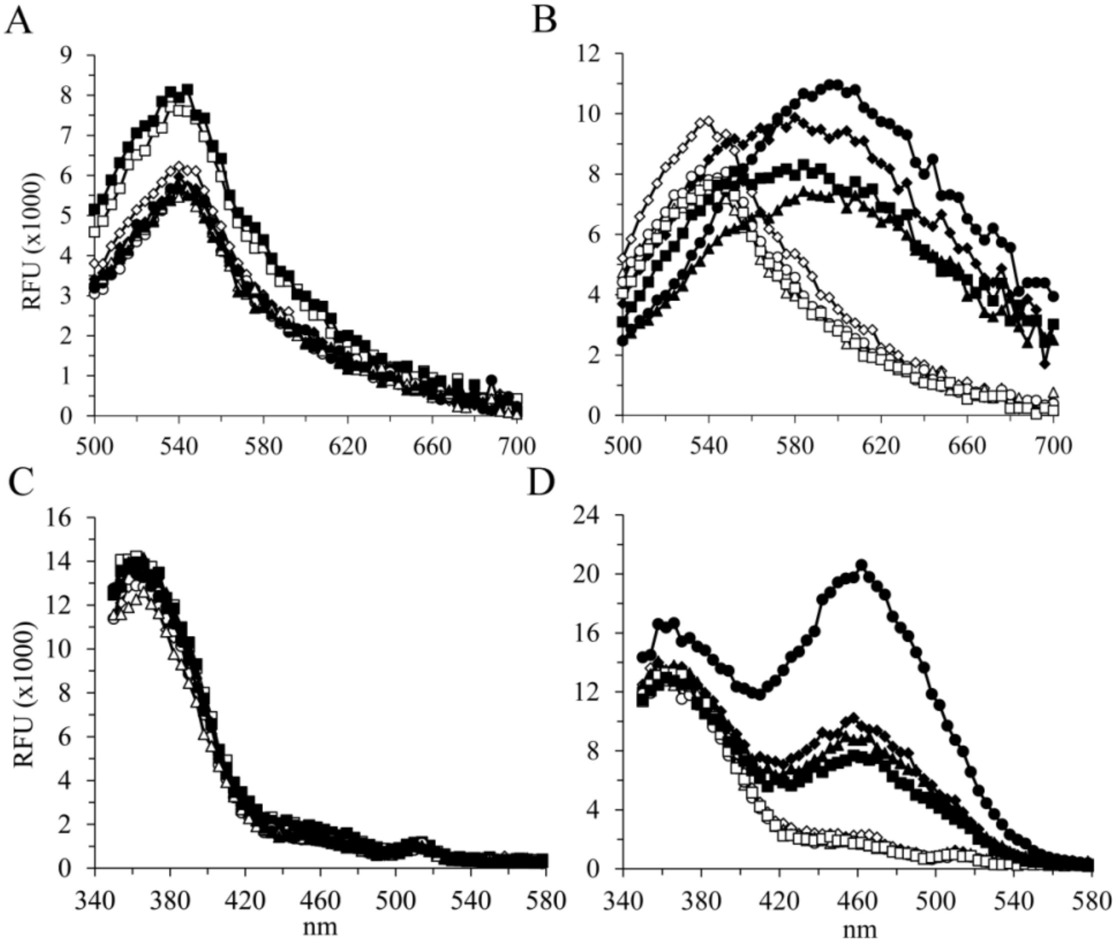

Fig. S6: Pregnancy plasma samples show the expected signals of HHPQ in the excitation and emission scan only after addition of cadaverine

Four healthy volunteer (**a, c**), and four representative 3rd trimester pregnancy (**b, d**) EDTA plasma samples were incubated with PBS (white symbols) or 400 µM cadaverine (black symbols) in duplicate for 1 h at 37°C. After TCA precipitation and pH adjustment to 4.0 emission scans after excitation at 460 nm (**a, b**) and excitation scans using emission at 620 nm (**c, d**) were performed. Healthy volunteer EDTA plasma samples contained less than 1 ng/ml DAO, which is below the detection limit of this fluorescent assay. The average DAO concentration of the four pregnancy samples was 158 ng/ml. The fluorescence signal in panels A and B at 540 nm is probably the Raman scattering peak of water.

Histamine Peculiarity

In 1959 Shore et al. published that histamine condensates with o-phthalaldehyde (OPT) and the resulting fluorescent molecules can be measured using excitation at 365 nm and emission at 444 nm, although the reaction is not specific and histamine or the fluorophore(s) must be isolated before fluorescence measurements [2]. Condensation of OPT with histamine at -20°C improved the sensitivity and also specificity [3]. Nevertheless, the structure of the fluorophore(s) was not equivocally determined [2,4,5].

Histamine can be measured with high sensitivity and specificity using the state of the art competitive histamine ELISA (IM2015) originally developed by Immunotech but now sold by Beckman Coulter. A recently developed homogeneous time-resolved fluorescence (HTRF) histamine assay (Cisbio) probably uses the same antibody. Both assays perform well in buffer matrices but measurements of plasma histamine show quite some variability at histamine concentrations > 100 nM or 11 ng/ml (unpublished data). Histamine oxidation can be measured using HRP/H_2O_2-coupling, but antioxidants will interfere. The antibody-based assays are also expensive, if they are used with many samples. Therefore, there is a moderate need for a sensitive (µM range) direct detection method of remaining histamine or imidazole acetaldehyde in complex matrices.

The fluorescence generated after fusion of histamine with oABA is not sensitive enough, although we detected some fluorescence after incubating histamine with oABA at -32°C for a few days (Figure S7C and S7F). Imidazole with or without DAO did not show any fluorescence signal (Figure S7D-F). Interestingly, imidazole acetaldehyde generated by incubating histamine with DAO did show fluorescence after being stored for several days at the three tested temperatures, with the highest fluorescence signal at -32°C (Figure S7F).

Histamine can exist in 4 tautomers/conformers: 2 imidazole tautomers (N^{tau}-H and N^{pi}-H; 80% and 20% in aqueous solution respectively; [6]) and 2 aminoethyl orientiations classified as trans and gauche forms [7]. Perhaps one of these forms is more favorable at temperatures below 0°C, improving structural rearrangements of the fluorophores. We did not find a similar description of the imidazole acetaldehyde conformers but it is possible that the aldehyde also forms the trans and gauche forms. These fluorescent data would be interesting, if the condensation of imidazole acetaldehyde with oABA were stronger, but it is approximately 30-fold lower after 5 days at room temperature compared to HHPQ with a detection limit of about 10 µM. The usage of different oABA derivatives [8] and incubation conditions might improve the detection of imidazole acetaldehyde. We are aware of only 1 publication which attempted to measure imidazole acetaldehyde, possibly because the aldehyde reacts with proteins and is therefore unstable as free form and very difficult to measure [9]. Additionally, imidazole acetaldehyde is likely very rapidly converted to the stable acetic acid, which is excreted in

urine with or without riboside conjugation and can be readily quantified using high-performance liquid chromatography coupled with electrospray mass spectrometry [10].

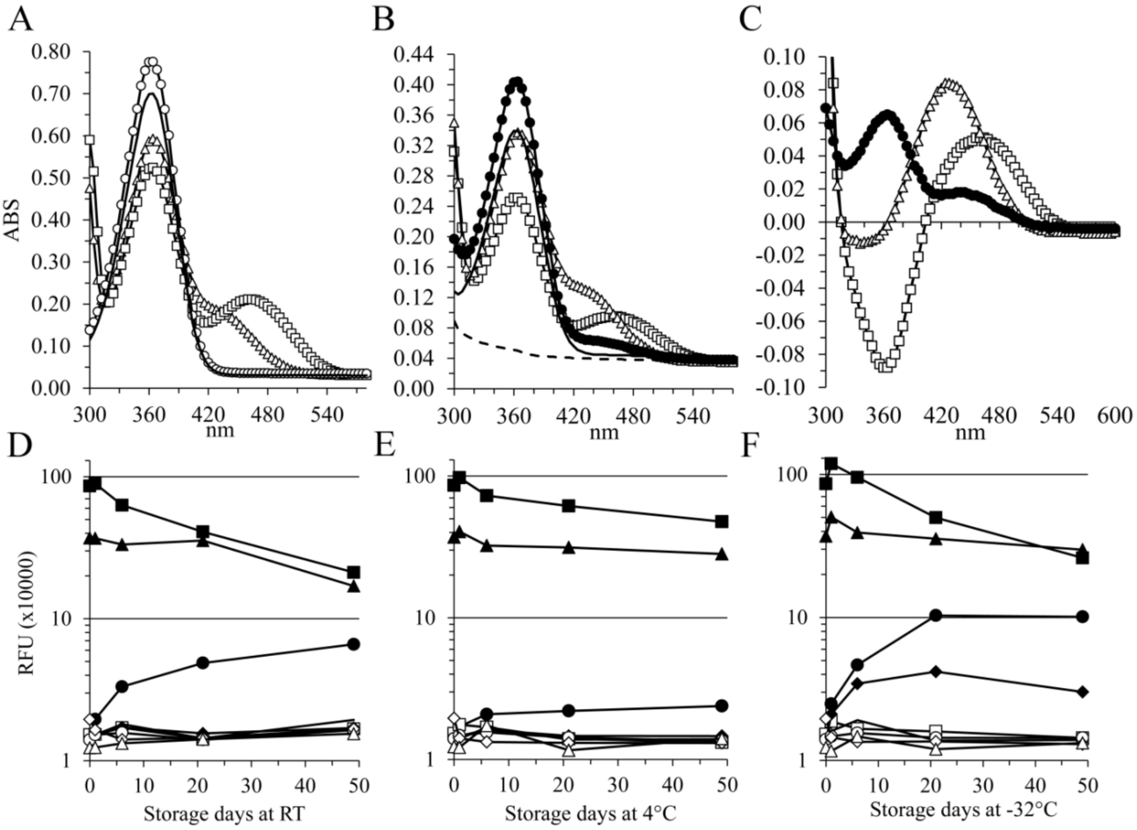

Fig. S7: Histamine and imidazole acetaldehyde condensate with oABA but absorption at ~440 nm and significant fluorescence develops only after several days of storage

(a) Fresh incubations of rhDAO with putrescine (white triangles), cadaverine (white squares), histamine (white circles) or just buffer (▬) and oABA result in the formation of THPQ and HHPQ. Imidazole acetaldehyde generated during incubation of rhDAO with histamine does not form a distinct chromophore but it likely condensates to some extent with oABA, because the oABA absorption peak at 360 nm is consistently higher (Panel A; data not shown) compared to just oABA. **(b)** After storage for 57 days at -32°C the same samples were remeasured and now histamine (black circles) incubated with DAO also shows a distinct absorption signal at ~450 nm; **(c)** Panel C shows the same data as in **(b)** after subtracting the buffer control absorption values; In a separate experiment we again synthesized THPQ and HHPQ but also tested histamine and imidazole without or with rhDAO and measured fluorescence five times over 50 days under three storage conditions: room temperature **(d)**, 4°C **(e)** or -32°C **(f)**; The following symbols are shown in Panels D, E and F: Black squares (■) HHPQ; black triangles (▲) THPQ; black circles (●) histamine with rhDAO; black diamonds (♦) histamine without rhDAO; white diamonds (◊) Hepes buffer without rhDAO; white circles (○) imidazole without rhDAO; white squares (□) imidazole with DAO; white triangles (Δ) Hepes buffer without oABA.

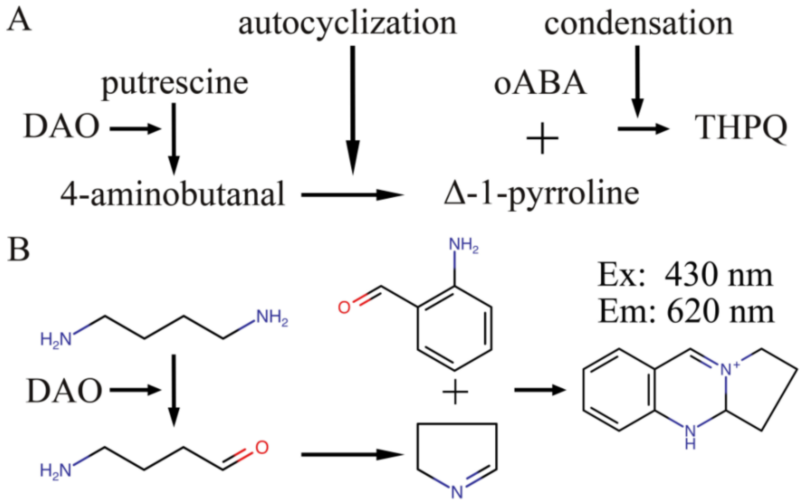

Fig. S8: Scheme for the generation of the quinazoline fluorophore THPQ from putrescine via DAO oxidation **(a)** After DAO oxidation of putrescine (butane-1,4-diamine) the released aldehyde 4-aminobutanal autocyclizes to delta-1-pyrroline (3,4-dihydro-2^{H}-pyrrole), which spontaneously condensates with ortho-aminobenzaldehyde (oABA) generating THPQ or 2,3,3a,4-tetrahydro-1^{H}-pyrrolo[2,1-^{b}]quinazoline-10-ium. The release of H_2O_2 and NH_3 during deamination, the release of H_2O during autocyclization and OH^- after condensation and rearrangement are not shown; (**b**) Chemical structures; Ex=Excitation; Em=Emission.

Supplement References

1. Schöpf C, Oechler F. Zur Frage der Biogenese des Vasicins (Peganins). Die Synthese des Desoxyvasicins unter physiologischen Bedingungen. Justus Liebigs Annal Chem. 1936;523:1–29. .

2. Shore PA, Burkhalter A, Cohn VH. A method for the fluorometric assay of histamine in tissues. J Pharmacol Exp Ther. 1959;127:182–6.

3. Håkanson R, Rönnberg AL. Improved fluorometric assay of histamine: condensation with O-phthalaldehyde at -20 degrees C. Anal Biochem. 1974;60:560–7. https://doi.org/10.1016/0003-2697(74)90267-x.

4. Rönnberg AL, Hansson C, Drakenberg T, Håkanson R. Reaction of histamine with o-phthalaldehyde: Isolation and analysis of the fluorophore. Anal Biochem. 1984;139:329–37.

5. Yoshimura T, Kaneuchi T, Miura T, Kimura M. Kinetic analysis of the fluorescence reaction of histamine with orthophthalaldehyde. Anal Biochem. 1987;164:132–7.

6. Ganellin CR. The tautomer ratio of histamine. J Pharm Pharmacol. 1973;25:787–92. https://doi.org/10.1111/j.2042-7158.1973.tb09942.x.

7. Vianello R, Mavri J. Microsolvation of the histamine monocation in aqueous solution: the effect on structure, hydrogen bonding ability and vibrational spectrum. New J Chem. 2012;36:954–62.

8. Richers MT, Deb I, Platonova AYu, Zhang C, Seidel D. Facile Access to Ring-Fused Aminals via Direct α-Amination of Secondary Amines with ortho-Aminobenzaldehydes. Synthesis of Vasicine, Deoxyvasicine, Deoxyvasicinone, Mackinazolinone and Ruteacarpine. Synthesis (Stuttg). 2013;45:1430–748.

9. Watanabe T, Kamijo K. A spectrophotometric assay of histaminase activity based on the hydrazone derivative of imidazole acetaldehyde with 2,4-dinitrophenylhydrazine. Chem Pharm Bull. 1977;25:185–91. https://doi.org/10.1248/cpb.25.185.

10. Kolmert J, Forngren B, Lindberg J, Öhd J, Åberg KM, Nilsson G, et al. A quantitative LC/MS method targeting urinary 1-methyl-4-imidazoleacetic acid for safety monitoring of the global histamine turnover in clinical studies. Anal Bioanal Chem. 2014;406:1751–62. https://doi.org/10.1007/s00216-013-7594-6.
